# Nitrate regulation of lateral root and root hair development in plants

**DOI:** 10.1093/jxb/erz536

**Published:** 2019-12-04

**Authors:** Bohan Liu, Junyu Wu, Shuaiqi Yang, John Schiefelbein, Yinbo Gan

**Affiliations:** 1 Zhejiang Key Laboratory of Crop Germplasm, Department of Agronomy, College of Agriculture and Biotechnology, Zhejiang University, Hangzhou, China; 2 Department of Molecular, Cellular, and Developmental Biology, University of Michigan, Ann Arbor, MI, USA; 3 Nanjing Agricultural University, China

**Keywords:** Lateral root, local signaling, long-distance communication, nitrate signaling, root foraging, root hair, systemic signaling

## Abstract

Nitrogen (N) is one of the most important macronutrients for plant growth and development. However, the concentration and distribution of N varies in soil due to a variety of environmental factors. In response, higher plants have evolved a developmentally flexible root system to efficiently take up N under N-limited conditions. Over the past decade, significant progress has been made in understanding this form of plant ‘root-foraging’ behavior, which is controlled by both a local and a long-distance systemic nitrate signaling pathway. In this review, we focus on the key components of nitrate perception, signaling, and transduction and its role in lateral root development. We also highlight recent findings on the molecular mechanisms of the nitrate systemic signaling pathway, including small signaling peptides involved in long-distance shoot–root communication. Furthermore, we summarize the transcription factor networks responsible for nitrate-dependent lateral root and root hair development.

## Teaser

This review provides an update on the molecular mechanisms involved in the long-distance shoot–root communication in systemic nitrate signaling, and outlines the transcription factor network responsible for nitrate regulation of lateral root and root hair growth.

## Introduction

Nitrogen (N) is one of the primary mineral nutrients for plant growth and development, as well as one of the main components of commercial fertilizers. N applications need to be repeated in order to maintain N availability and soil fertility (Mallory *et al.*, 2010). Nitrate (NO_3_^–^) and ammonium (NH_4_^+^) are the main mineral forms of N that plants utilize from their external environment, but the fluctuating environment and the intrinsic complexity of soils cause numerous reactions, transformations, and N losses that generate tremendous variation in soil N distribution (Zhu *et al.*, 2011; Ye *et al.*, 2015; Berhe and Torn, 2017; Pandey *et al.*, 2019). The highly plastic plant root system is able to respond developmentally to the N nutrient signal, enabling exploration and colonization into N-rich patches of soil. However, crops are only able to use 30–50% of the applied N in general. Therefore, an understanding of the mechanism of nitrate-regulated lateral root (LR) and root hair development may lead to increased nitrogen use efficiency (NUE) in crops (Bowles *et al.*, 2018).

The plant root system is not only regulated by the ‘N signal’, which represents the autonomous response to local nitrate availability (Remans *et al.*, 2006), but it is also affected by the so-called ‘systemic N signal’ pathway, which selectively promotes colonization of plant roots in N-rich patches (Bellegarde *et al.*, 2017; Ohkubo *et al.*, 2017). Recent studies demonstrated that nitrate-dependent root foraging is controlled by complex interactions between nitrate perception, signaling, and the systemic regulatory pathways. This review will summarize our understanding of these interactions and provide an outline of shoot–root communication in nitrate systemic regulation. In addition, we describe current progress in discovering the genetic network for nitrate-dependent LR and root hair regulation.

## Lateral root and root hair development response to external nitrate

The root systems of higher plants have evolved to be highly plastic, capable of recognizing and colonizing fertile soils; a phenomenon known as ‘root foraging’ (Drew *et al.*, 1973; Motte and Beeckman, 2019). In general, root foraging is triggered by an uneven distribution of nutrients in the environment. For example, a localized concentration of nitrate, as compared with uniform nitrate levels, promotes more significant root development changes in plants (Zhang and Forde, 2000). Such a localized external nitrate treatment could stimulate LR development in many plant species, including maize, barley, rice, and Arabidopsis (Xu *et al.*, 2012; Forde, 2014). In homogenous nitrate conditions, N deficiency (<1 mM) represses LR development, and excessive N supply (>10 mM) exerts a systemic inhibitory effect on LR development (Zhang *et al.*, 1999, 2007). It is interesting that the post-embryonic LRs rather than the embryonic primary root or seminal root tend to show more sensitivity and plasticity in response to external nitrate signals in both dicots and monocots, despite the substantial differences in monocot and dicot root system architecture (Forde, 2014; Tian *et al.*, 2014; Steffens and Rasmussen, 2016).

**Fig. 1.**
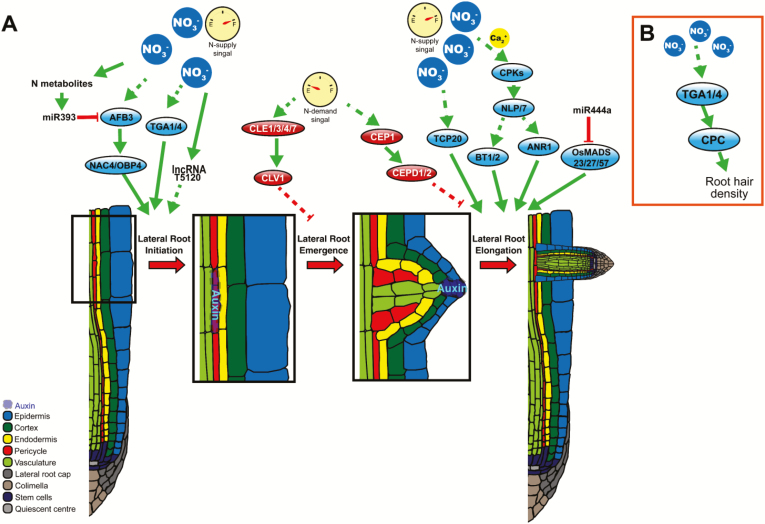
Schematic presentation of the genetic network regulating LR and root hair development in plants. (A) Schematic presentation of the genetic network regulating LR development: LR initiation, LR emergence, and LR elongation. (B) Schematic presentation of the genetic network regulating root hair development. Blue ovals indicate positive regulators. Red ovals indicate negative regulators. Green arrows indicate the positive regulation, and red lines indicate negative regulation.

In a typical dicot tap root system, such as Arabidopsis, the embryonic primary root grows vertically downward, and smaller post-embryonic LRs arise from the sides (Petricka *et al.*, 2012). In monocot plants, such as rice, wheat, and maize, their fibrous root systems contain embryonic primary and seminal roots, as well as post-embryonic adventitious roots (also called nodal roots, brace roots, or crown roots) and LRs (Coudert *et al.*, 2010; Hochholdinger *et al.*, 2018). Despite the major divergence of root system architecture between monocots and dicots, the formation of LRs seems to be fairly conserved at the anatomical level (Motte and Beeckman, 2019). LR development begins from founder cells in the pericycle tissue; these founder cells divide to form an LR primordium, and finally the developing LR primordium emerges from the epidermis and becomes the newly formed LR (Peret *et al.*, 2009). The subsequent elongation of LRs is regulated by both external and internal stimuli (Fig. 1A) (Zhang *et al.*, 1999; Motte and Beeckman, 2019).

By utilizing a segmented agar plate technique or split-root system, the local nitrate effect on LR initiation, emergence, and elongation has been studied. In non-legume dicot plants, such as *Arabidopsis thaliana* and tomato, the local nitrate treatment mainly affects the elongation of LRs, and has little or no effect on LR numbers (Zhang *et al.*, 1999; Linkohr *et al.*, 2002; Lu *et al.*, 2010; Forde, 2014), except that a local nitrate deprivation signal does suppress LR initiation in Arabidopsis (Linkohr *et al.*, 2002). In legume dicots, such as *Medicago truncatula* and *Phaseolus vulgaris*, nitrate not only promotes growth of LRs but also affects root nodule formation (Guo *et al.*, 2007; Ruffel *et al.*, 2008). On the other hand, in monocots (such as cereals), the local nitrate effect displays a more complicated behavior as LRs originate from different types of roots. For example, both the initiation and elongation of LRs could be induced by local nitrate supply in barley and wheat (Hackett, 1972; Drew *et al.*, 1973). However, in 7- or 14-day-old rice seedling, local nitrate supply only affected elongation of LRs on primary and seminal roots, and LR number was not changed (Wang *et al.*, 2002). In 7-day-old maize seedlings, only elongation of LRs on primary roots is induced (P. Yu *et al.*, 2014), but older maize seedlings show alterations in the density and length of LRs on crown roots by local nitrate treatment (in ‘t Zandt *et al.*, 2015). Thus, the LRs of various cereal species respond differently to nitrate, implying that both species-specific and developmental stage-specific effects are involved. Further research into these differing mechanisms will be essential for better understanding of the nitrate-triggered LR-foraging phenomenon.

Root hairs are specialized epidermal cells that participate in water and nutrient uptake. Surprisingly, there are only few reports about the effect of nitrate availability on root hair development (Foehse and Jungk, 1983; Robinson and Rorison, 1987; Vatter *et al.*, 2015). In tomato, spinach, and rape, the density and length of root hairs were reported to be negatively correlated with homogenous nitrate availability (Foehse and Jungk, 1983). In four grass species, the root hair density and length were also reported to decrease as the nitrate concentration increases in nutrient solution (Robinson and Rorison, 1987). These data indicate that, in a homogenous nitrate environment, root hair development is gradually inhibited by a systemic effect similar to the effect on LR development. Interestingly, in a recent report, root hair development in Arabidopsis was found to respond to local nitrate availability (Alvarez *et al.*, 2014; Vatter *et al.*, 2015). In Arabidopsis, root hair density generally increased with local nitrate concentration in different ecotypes (Vatter *et al.*, 2015). These results indicate the root hair development, like LR development, is under the control of both local and systemic nitrate signaling effects.

## Regulation of lateral roots and root hair development by a nitrate-responsive regulatory network

Nitrate serves not only as an N source but also as a signaling molecule in plant nutritional response (Wang *et al.*, 2007). In Arabidopsis, the nitrate signaling pathways have been studied intensively. External nitrate is initially perceived by the dual-affinity transceptor *AtNRT1.1*/*CHL1*/*AtNPF6.3* (*AT1G12110*) (Tsay *et al.*, 1993; Liu *et al.*, 1999). After perception, the signal is further transduced by a calcium-dependent pathway and a calcium-independent pathway (Riveras *et al.*, 2015; Zhang *et al.*, 2019). In the calcium-dependent pathway, calcium acts as a secondary messenger for the nitrate signal. Its accumulation in the cytoplasm and nucleus accompanies the nuclear translocation of CALCIUM-SENSOR PROTEIN KINASES (CPKs), such as AtCPK10, AtCPK30, and AtCPK32 (Liu *et al.*, 2017). The nuclear translocation of AtCPK10/30/32 transduces the signal into the regulatory networks in the nucleus, triggers the primary nitrate response, and further influences the development of the root system (Liu *et al.*, 2017). In the calcium-independent pathway, the expression of *AUXIN SIGNALING F-BOX3* (*AtAFB3*) is triggered by intracellular nitrate, and this regulates downstream genes such as *NAC3/OBP4* that influence primary root and LR development (Vidal *et al.*, 2010, 2013; Riveras *et al.*, 2015).

Although nitrate perception and primary nitrate response is a rapid and local process that takes a few minutes, the signal needs to be further transmitted to initiate the physiological and developmental response (Wang *et al.*, 2003; Hu *et al.*, 2009). These kinds of response to nitrate, such as the regulation of root system development, are much slower, usually needing hours or days. The transcription factors involved in nitrate-dependent LR development collaborate with both local and systematic signals, and these transcription factors are discussed below (Fig. 1A, B).

### The nitrate–CPK–NLP signaling plays a central role in nitrate-responsive lateral root regulation

Ca^2+^ is an universal second messenger of diverse signaling pathways, involved in biotic and abiotic stresses (Knight *et al.*, 1996; Boudsocq *et al.*, 2010). By introducing aequorin reporter lines, the levels of calcium were observed to fluctuate in cytoplasm in response to nitrate availability. In addition, this process occurs downstream of *AtNRT1.1*, which indicates that Ca^2+^ can serve as a second messenger in nitrate signaling (Riveras *et al.*, 2015). On the other hand, application with a phospholipase C (PLC) inhibitor (U73122), calcium chelator EGTA, or the calcium channel blocker La^3+^ could also repress the nitrate-induced expression of *NIR* and *NIA* genes in Arabidopsis, maize, and barley (Sakakibara *et al.*, 1997; Sueyoshi *et al.*, 1999; Riveras *et al.*, 2015). These data demonstrated that the PLC activity and Ca^2+^ play a crucial role in nitrate signaling. In the Arabidopsis chemically engineered mutant *icpk*, mutation of *CPK10*, *CPK30*, and *CPK32* disrupts not only the nitrate-specific stimulation of LR initiation and elongation, but also the nuclear location and phosphorylation of NIN-like proteins (NLPs; Liu *et al.*, 2017). Further studies show that CPK–NLP signaling controls both the primary nitrate response and nitrate-responsive root regulation (Castaings *et al.*, 2009; Liu *et al.*, 2017). In Arabidopsis, AtNLP7 was reported to be capable of binding to the promotor region of several nitrate-responsive transcription factors, such as *ANR1* and *BT1/2* (Marchive *et al.*, 2013). In legume species, *NRSYM1/LjNLP4* in *Lotus japonicus* and *MtNLP1/4* in *M. truncatula* were shown to be involved in nitrate-dependent nodule formation (Lin *et al.*, 2018; Nishida *et al.*, 2018). Combined together, the nitrate–CPK–NLP signaling pathway may be genetically conserved in the nitrate response regulatory network in most plants, thereby influencing LR development and nodule formation (Liu *et al.*, 2017; Mu and Luo, 2019).

### miR393/AFB3 module involved in systemic regulation of lateral root initiation

In the Arabidopsis calcium-independent nitrate signaling pathway, elevated intracellular nitrate induces expression of *AFB3* in root tips and the pericycle area (Vidal *et al.*, 2010; Riveras *et al.*, 2015). Mutation of *AFB3* leads to shorter primary roots and lower LR density in the nitrate-rich side of a split-root system, which indicates that *AFB3* is involved in regulating nitrate-dependent LR initiation and primary root development (Vidal *et al.*, 2010). Further studies revealed that *AFB3* acts upstream of *NAC3* and *OBP4* by up-regulating the expression of *NAC3* and *OBP4* to further control LR initiation under auxin signaling (Vidal *et al.*, 2013). In response to a systemic signal, the AFB3 module could be feedback regulated by *AtmiR393*, an miRNA induced by products of nitrate assimilation (Vidal *et al.*, 2010). Working together, *AtmiR393/AFB3*, *NAC3*, and *OBP4* are involved in the crosstalk between nitrate signaling and auxin signaling to regulate LR initiation (Fig. 1A).

### The role of AGL17-Like MADS-box transcription factor in nitrate-dependent regulation of lateral root elongation

In Arabidopsis, the AGL17-Like gene *AGL44*/*AtANR1* was first reported as a MADS-box transcription factor, involved in promoting LR elongation in response to localized nitrate treatment (Zhang and Forde, 1998; Gan *et al.*, 2012). *AtANR1* is expressed in LR primordia and the primary root apex, it promotes meristematic activity, and it was induced by nitrate signal downstream of *AtNRT1.1* (Remans *et al.*, 2006). Overexpression of *AtANR1* significantly increased the LR number and length in Arabidopsis (Gan *et al.*, 2012). In rice, there are five AGL17-Like homologs (*OsMADS23*, *25*, *27*, *57*, and *61*), and three of them (*OsMADS25*, *27*, and *57*) were responsive to nitrate signal (C.Y. Yu *et al.*, 2014). *OsMAD25* and *27* exert an inhibitory effect on primary root development, but they promote the elongation of LRs in an auxin-dependent manner (Yu *et al.*, 2015; Chen *et al.*, 2018; Zhang *et al.*, 2018). Additionally, the overexpression of *OsMAD25* and *27* enhances salinity tolerance in rice via modulation of the ABA signaling pathway (Chen *et al.*, 2018; Xu *et al.*, 2018). The monocot-specific miR444 affects post-transcription inhibition of OsMADS genes, because overexpression of *OsmiR444* reduced the accumulation of *OsMADS23*, *27*, and *57* which in turn decreased LR elongation in a nitrate-dependent manner (Yan *et al.*, 2014). Additionally, the AGL17-Like homolog in chrysanthemum, *CmANR1*, had also been reported to positively modulate both adventious root and LR development, which occurs by directly regulating the auxin transport gene *CmPIN2* (Sun *et al.*, 2018). Despite the evolutionary divergence between these species, the role of ANR1-related genes seems to be conserved for regulating nitrate-dependent LR elongation in plants (Fig. 1A).

### BT1/2 is involved in systemic regulation of lateral root elongation

In Arabidopsis, the *Bric-a-Brac/Tramtrack/Broad (BTB) and TAZ DOMAIN PROTEIN 2* (*BT2*) was first found as an activator of telomerase in mature leaves, and it also plays a crucial role in gametophyte development (Ren *et al.*, 2007). Further studies have demonstrated that the expression of *BT2* is under the control of light, nutrients, hormones, and stresses, suggesting that BT2 is a key element in integrating multiple signaling networks (Mandadi *et al.*, 2009). Using systems biology and bioinformatic tools, BT2 has been predicted to be the potential regulator in the NUE network (Araus *et al.*, 2016). *BT1* is the closest homolog of *BT2*, and expression of both *BT1* and *BT2* is nitrate inducible (Sato *et al.*, 2017). Under low nitrate availability conditions, the *bt1/bt2* double mutant exhibited an extended LR phenotype as compared with wild-type plants (Araus *et al.*, 2016). However under high nitrate conditions, the *bt1-1 bt2-4* double mutant showed shorter LRs in comparison with wild-type plants (Sato *et al.*, 2017). These results suggest that *BT1* and *BT2* are required not only for LR elongation under high nitrate conditions, but also for the inhibition of LR elongation under low nitrate availability. The distinct roles played by *BT1* and *BT2* under different nitrate conditions implicates them in systemic regulation of LR development (Fig. 1A).

### TCP20 interacts with NLP6/7 to control systemic regulation of LR elongation

In Arabidopsis, by studying the nitrate-responsive *cis*-element (NRE) region of *NRT2.1* and *NIA1* using the yeast one-hybrid assay, the *TEOSINTE BRANCHED1/CYCLOIDEA/PROLIFERATING CELL FACTOR1-20* (*AtTCP20*) was identified as binding the promotor of *NRT1.1*, *NRT2.1*, and *NIA1* (Guan *et al.*, 2014). Mutation of *AtTCP20* causes defective nitrate foraging root phenotypes in split-root systems, indicating that AtTCP20 plays a key role in the systemic nitrate response (Guan *et al.*, 2014). Further studies show the AtTCP20 physically interacts with AtNLP6/7, and subcellular localizations of TCP20/NLP6/7 complexes depend on nitrate availability (Guan *et al.*, 2017). The intranuclear interaction between AtTCP20 and AtNLP6&7 represses expression of the cell cycle marker gene *CYCB1;1* in roots under N starvation (Guan *et al.*, 2017). In other species, such as chrysanthemum, the overexpression of *CmTCP20* influences auxin accumulation and promotes LR development (Fan *et al.*, 2019*a*, *b*). Further studies show that CmTCP20 also interacts with CmARF8, binding to the proximal site in the promoter region of *CmCYCB1;1* to positively regulate the cell cycle in the root (Fan *et al.*, 2019*b*). These results indicate that *TCP20* has two distinct functions in root development under the control of local and systemic signaling, and may be genetically conserved in plants (Fig. 1A).

### TGA1 and TGA4 respond to local nitrate and regulate LR development and root hair initiation

By integrative network bioinformatics, *TGA1* and *TGA4* were identified as potential regulatory factors of nitrate response in Arabidopsis (Alvarez *et al.*, 2014). Expression of both *TGA1* and *TGA4* is induced downstream of AtNRT1.1 with external nitrate application (Alvarez *et al.*, 2014). ChIP assays revealed that TGA1 could directly bind to the promoter of *AtNRT2.1/2.2* and promote the expression of *NRT2.1/2.2* (Alvarez *et al.*, 2014). Mutations of both *TGA1* and *TGA4* inhibit LR initiation, emergence, root hair initiation, and primary root length (Alvarez *et al.*, 2014; Canales *et al.*, 2017). These results indicate that the *TGA1/4* genes regulate LR and root hair initiation by acting downstream of *AtNRT1.1* but upstream of *AtNRT2.1/2.2* (Fig. 1A) (Alvarez *et al.*, 2014). However, only a few studies revealed that root hair development also responds to nitrate levels. Nitrate was reported to play a key role in controlling root hair initiation on developing roots (Canales *et al.*, 2014, 2017; Vatter *et al.*, 2015). The meta-transcriptomics analysis demonstrated that a set of co-expressed genes involved in root hair development also respond to external nitrate (Canales *et al.*, 2014). Similar to LR development, root hair density of the NRT1.1-related mutant displayed a significant reduction in comparison with wild-type plants, suggesting that root hair development is also controlled by NRT1.1-based nitrate signaling (Vatter *et al.*, 2015; Canales *et al.*, 2017). Further experiments revealed that external nitrate treatment could increase root hair density in Arabidopsis. The increased root hair number is mainly due to a reduction in the longitudinal cell length of trichoblasts, and this process requires *NRT1.1*, *TGA1/4*, and *CPC* (Canales *et al.*, 2017). The ChIP assay demonstrated that TGA1 could directly bind to the –1839 to –1831 region of the *CPC* promoter, which promotes *CPC* expression (Canales *et al.*, 2017). These results suggest that a TGA1/CPC module responds to an NRT1.1-mediated external nitrate signal to control root hair density and nitrate uptake efficiency in Arabidopsis (Fig. 1B).

### A CLE–CLV1 module involved in systemic lateral root inhibition

The CLAVATA3 (CLV3)/ENDOSPERM SURROUNDING REGION (ESR)-related (CLE) family were first identified as extracellular peptides that interact with CLAVATA1 (CLV1), a type XI leucine-rich repeat receptor-like kinase (LRR-RLK), to control cell proliferation and differentiation in the shoot apical meristem in Arabidopsis (Fiers *et al.*, 2007; Butenko *et al.*, 2009). Among 32 CLE genes in Arabidopsis, the expression of four CLE genes (*CLE1*, *3*, *4*, and *7*) was found to be induced in a dose-dependent manner in roots under nitrate deficit conditions (Araya *et al.*, 2014; Goad *et al.*, 2017). Promoter-driven green fluorescent protein (GFP) analyses indicate that *CLE1*, *2*, *3*, *4*, and *7* are predominantly expressed in root pericycle cells, and overexpression of any of these genes is sufficient to inhibit LR development (Araya *et al.*, 2014). On the other hand, among mutants of the nine type XI LRR-RLK genes, only the *clv* mutant displayed a significant extension of LRs under N deficit conditions (Araya *et al.*, 2014). Overexpression of *CLE3* leads to significant LR inhibition, but not in the *clv1-4* mutant background, which indicates that CLE-induced LR inhibition requires CLV1 (Araya *et al.*, 2014). *CLV1* expression is located in phloem companion cells, separate from pericycle cells expressing *CLE3*, implying that the CLE–CLV1 module participates in intercellular signaling for the systemic N response. The CLV3/ESR (CLE) protein family is found in at least 19 species across monocots and dicots (Oelkers *et al.*, 2008). In addition to data in Arabidopsis, the CLEs were reported to be involved in root nodulation of legumes and root development in wheat (Mortier *et al.*, 2010; Nishida *et al.*, 2018; Li *et al.*, 2019). The above evidence implies that CLE function in nitrate-dependent root regulation may be conserved in other species.

### Long non-coding RNA T5120 regulates nitrate-dependent lateral root initiation

In Arabidopsis, *TCONS_00005120(T5120)* was identified as a novel long non-coding RNA (lncRNA) induced by external nitrate, using high-throughput strand-specific RNA-seq (Liu *et al.*, 2019). The expression of *T5120* was very low in all tested organs, and it was strongly induced by nitrate in a time- and concentration-dependent manner (Liu *et al.*, 2019). In an NR-null mutant (*nia1 nia2*), the expression of *T5120* could still be specifically induced by external nitrate, meaning that *T5120* responds to nitrate itself but not its reduction products (Liu *et al.*, 2019). Further experiments demonstrated that AtNLP7 directly binds to the NRE-like motif of the T5120 promoter and positively regulates *T5120* transcription (Liu *et al.*, 2019). The lncRNA T5120 acts downstream of AtNLP7 and AtNRT1.1 and promotes nitrate assimilation and LR initiation in Arabidopsis (Liu *et al.*, 2019). The lncRNAs have been demonstrated to play multiple roles in plant development, but given that lncRNAs are highly variable during evolution, the functions of lncRNAs in nitrate-dependent processes need to be further investigated in other species (Ulitsky, 2016). Thus, screening potential lncRNAs involved in nitrate-dependent root development could be a practical tool for improved NUE in crops.

## Distance communication involved in nitrate-dependent root regulation

**Fig. 2.**
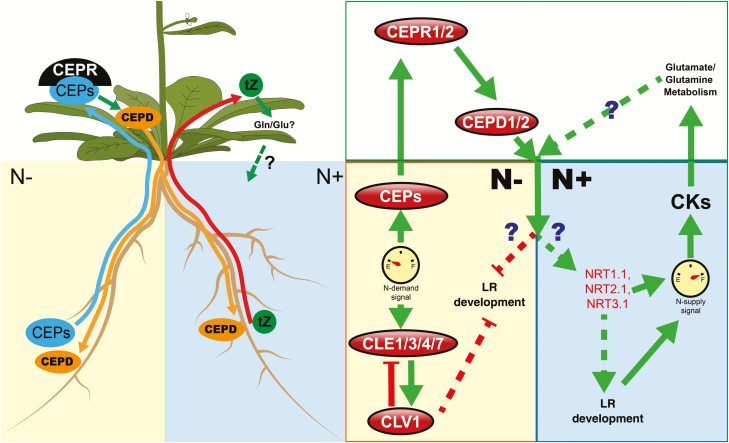
Schematic presentation of distance communication in nitrate systemic signaling in plants. Left: distance communication in nitrate systemic signaling. The blue arrow indicates the ascending N-demanding signal, the red arrow indicates the ascending N supply signal, yellow arrows indicate the descending signal, and green arrows indicate positive regulation. Right: schematic presentation of the genetic network involved in nitrate systemic signaling. A green oulined square represents the shoots. The orange outlined square represents the N-deprived side of of root. The blue outlined square represents the N-rich side of the root. Green arrows indicate positive regulation, and red lines indicate negative regulation.

The root system represents the only subterranean portion of the plant, and recent studies show that the complicated root-foraging response depends on long-distance root–shoot–root communication (Ruffel *et al.*, 2011; de Bang *et al.*, 2017; McCleery *et al.*, 2017; Ohkubo *et al.*, 2017; Roy, 2018). These studies suggest that N demand signals are generated from the N-deprived side of the root system, and are transmitted from root to shoot (Ohkubo *et al.*, 2017). On the other hand, N supply signals are generated from the N-rich side of the root system and are also transmitted from root to shoot (Ruffel *et al.*, 2011). The shoot perceives these N demand versus N supply signals and, in turn, generates corresponding signals that are transmitted to each side of the root system to modulate root development, *NRT2.1* expression, and nitrate uptake (Fig. 2).

### A CEP1–CEPR1/2–CEPD1/2 small peptide hormone pathway is required for N demand signaling

The *C-TERMINALLY ENCODED PEPTIDE* genes were discovered using an *in silico* approach. They are post-translationally modified, secreted peptide hormones that respond to N demand signaling, and they act through their receptors to control nodulation and root architecture in plants (Taleski *et al.*, 2018). In Arabidopsis, C-terminally encoded peptide 1 (AtCEP1) was first reported as a small peptide hormone expressed in LR primordia to function in inhibition of root growth (Ohyama *et al.*, 2008). *In silico* analyses confirmed a total of 15 CEP genes in the Arabidopsis genome, including seven (*AtCEP1*/*3*/*5*/*6*/*7*/*8*/*9*) up-regulated when roots suffer from N starvation conditions. These small peptides are synthesized in the stele of LRs (Roberts *et al.*, 2013; Tabata *et al.*, 2014), and then loaded into the xylem vessels and further transported from root to shoot as an ascending systemic N demand signal to leaves (Tabata *et al.*, 2014). In vascular tissues of the leaf, AtCEP1 is perceived by an LRR-RK, named CEP receptor 1/2 (AtCEPR1/2) (Tabata *et al.*, 2014). Mutating both *AtCPER1* and *AtCEPR2* not only caused growth retardation in N-rich medium with N deficiency symptoms, but also impaired the systemic N demand signaling response (Tabata *et al.*, 2014). When shoots perceived the CEP1 signal from roots by AtCEPR1 and AtCEPR2, the non-secreted small signaling peptides AtCEPD1 and AtCEPD2 were induced and then translocated to both N-rich and N-deprived sides of the root. However, only the expression of *NRT2.1* in the N-rich side of LRs was induced (Ohkubo *et al.*, 2017). These results suggest that the descending AtCEPD1/2 signal needs to cooperate with local signals on both sides (Ohkubo *et al.*, 2017). On the other hand, the *cepd1,2* double mutant produces longer LRs, which indicates that the descending AtCEPD1/2 signal might be involved in systemic inhibition of LR elongation in the nitrate-deprived side (Fig. 2) (Ohkubo *et al.*, 2017).

In other species, the CEPs were also reported to be involved in nitrate-dependent root development. In *M. truncatula*, the expression of* MtCEP1/2* was up-regulated under low nitrate conditions, inhibited LR initiation and emergence, and promoted nodule formation (Imin *et al.*, 2013). In *Oryza sativa*, the expression level of *OsCEP6.1* was induced by low nitrate availability. The overexpression of *OsCEP6.1* and application of synthesized OsCEP6.1 significantly reduced root growth (Sui *et al.*, 2016). In addition, phylogenetic analyses indicated that CEP genes exist in most angiosperm plants, which means that CEP-mediated pathways may be conserved during plant evolution (Ogilvie *et al.*, 2014). Together, these studies suggested that CEP peptide hormones play important roles in orchestrating N demand signaling, root nodulation, and LR development in plants.

### Root–shoot communication of cytokinin involved in N supply systemic signaling

Among phytohormones, cytokinin (CK) has been considered to be closely linked to N signaling (Miyawaki *et al.*, 2004; Sakakibara *et al.*, 2006). Previous studies demonstrated that the CK-dependent systemic N demand signaling controls LR initiation in Arabidopsis (Ruffel *et al.*, 2016). Recently, by introducing *abcg14* and the isopentenyl transferase *ipt3 ipt5 ipt7* triple mutant to the typical split-root system, CK was confirmed to play an essential role in root to shoot communication of systemic N demand signaling (Ruffel *et al.*, 2011; Poitout *et al.*, 2018). Compared with wild-type plants, the *ipt3 ipt5 ipt7* triple mutant failed to maintain a proper primary response in the split-root system, which means that biosynthesis of CK is required for N systemic signaling under heterogeneous nitrate conditions (Poitout *et al.*, 2018). Similarly, the *abcg14* mutant, which cannot translocate CKs from root to shoot, also displayed an impaired systemic N response phenotype (Poitout *et al.*, 2018). A UHPLC-MS analyses of various active forms of CK in shoots and roots revealed that *trans*-zeatin (tZ) accumulation in shoots is required for the systemic N response in roots (Poitout *et al.*, 2018). Collectively, the biosynthesis of CKs was triggered by N-rich conditions in roots, then the tZ-type CK was translocated to the shoot by ABCG14 as an ascending signal, causing accumulation of active tZ in shoots that controls the N supply signaling in roots (Fig. 1) (Takei *et al.*, 2004; Ko *et al.*, 2014; Zhang *et al.*, 2014; Poitout *et al.*, 2018). Transcriptomic analyses indicate that the translocated active tZ in shoots results in modified expression of glutamate and glutamine biosynthesis (Fig. 2) (Poitout *et al.*, 2018). This further confirms the importance of CK-dependent root–shoot communication in nitrate systemic signaling, and leads to a model in which CK-dependent root–shoot communication affects glutamate/glutamine metabolism in shoots, emphasizing the role of CK in nitrate systemic signaling.

## Concluding remarks and future perspectives

Over the past several years, an increasing number of players involved in local and systemic nitrate signaling have been discovered. The emerging story of nitrate-dependent systemic regulation places particular emphasis on root–shoot–root distance communication (Ohkubo *et al.*, 2017; Poitout *et al.*, 2018). After external nitrate signals are perceived by roots, a signal is generated that travels from the root to the shoot. In turn, the shoots produce a descending signal responsible for the root-foraging phenomenon. However, we still lack an understanding of how the descending shoot–root signals act differently on each side of split-root systems (Ohkubo *et al.*, 2017). Furthermore, the nitrate-dependent LR development is clearly under control of both local and systemic nitrate signaling pathways. Since the systemic signaling is a new and emerging field of nitrate signaling, we have highlighted some of the key components responsible for root foraging in both local and systemic signaling pathways. For example, the CEPD1/2 descending signal is distributed equally, but it causes different phenotypes under different nitrate conditions (Ohkubo *et al.*, 2017). Further, the transcription factors TGA1/4, TCP20, and BT1/BT2 were shown to have essential roles in systemic nitrate signaling, but we still have little mechanistic understanding of the crosstalk between the local and systemic nitrate signaling pathways (Guan *et al.*, 2014; Araus *et al.*, 2016; Sato *et al.*, 2017). There is a need to use integrated systems biology approaches and functional genomics tools to dissect the complex regulatory networks behind these processes.

At the same time, many regulators have been identified that influence LR development in the nitrate-dependent signaling pathway in model plants, such as Arabidopsis and rice (Vidal *et al.*, 2010; Guan *et al.*, 2014; Wang *et al.*, 2018). The LR initiation determines the number of LRs that branch from the primary root, while the elongation of LRs enhances nitrate uptake (Zhan and Lynch, 2015). Moreover, root hair development is also regulated by nitrate (Vatter *et al.*, 2015). As shown in Fig. 1, there are multiple regulators responsible for the same process, implicating that these regulatory mechanisms are redundant, and in some cases antagonistic to each other. Thus, the genetic relationship between the various regulators still remains elusive and requires the use of an array of molecular and genetic tools to decipher. The nitrate-dependent regulation of LR and root hair development may share a common intrinsic mechanism (such as NLPs, AGL14-like MADS-box transcription factors, and TCP20), but some of the regulators (such as OsmiR444 and lncRNA T5120) are species specific. Considering that most of these regulator effects on nitrate-dependent LR and root hair development have only been reported in Arabidopsis, more species need to be tested for these regulators in order to dissect the specificity or generality of the regulatory pathways. Although there is increased understanding of nitrate signaling in grasses, it will be important to further investigate any potential new regulatory mechanisms in more species. Progress in understanding nitrate signaling and regulation mechanisms in monocot crops will probably contribute to the development of sustainable and energy-efficient agriculture.

## Abbreviations:

ABCG: ATP-binding cassette subfamily G
AFB: auxin signaling F-box
ANR1: Arabidopsis nitrate regulated 1
BT2: Bric-a-brac/Tramtrack/Broad (BTB) and TAZ domain protein 2
CBL: calcineurin B-like protein
CEP: C-terminally encoded peptide
CEPD: CEP downstream
CEPR: CEP receptor
CHL: chlorate
CK: cytokinin
CLE: CLAVATA3/endosperm surrounding region (ESR)-related
CLV: CLAVATA
CPC: CAPRICE
CPK: calcium sensor protein kinase
GFP: green fluorescent protein
IPT: isopentenyl transferase
LR: lateral root
LRR-RLK: leucine-rich repeat receptor-like kinase
MADS: MINICHROMOSOME MAINTENANCE1/AGAMOUS/DEFICIENS/SERUM RESPONSE FACTOR
NAC: NAM/ATAF/CUC
NBIP1: NRT1.1B-interacting protein 1
NIA: nitrate reductase
NIN: NODULE INCEPTION
NIR: nitrite reductase
NLP: NIN-like protein
NPF: nitrate transporter 1/peptide transporter family
NRT: nitrate transporter
NUE: nitrogen use efficiency
OBP: OCS element-binding factor (OBF)-binding protein
TCP: TEOSINTE BRANCHED1/CYCLOIDEA/PROLIFERATING CELL FACTOR
TGACG: sequence-specific binding protein
tZ: *trans*-zeatin
